# An Intriguing Endoscopic Case of Asymptomatic Crohn’s Disease

**DOI:** 10.1177/2324709613503314

**Published:** 2013-09-05

**Authors:** Mena Boules, Ilyssa O. Gordon, Brian Kirsh, Maged K. Rizk

**Affiliations:** 1 Cleveland Clinic, Cleveland, OH, USA

**Keywords:** asymptomatic Crohn’s disease, endoscopic case of Crohn’s disease

## Abstract

We present the case of a 64-year old male with Crohn’s disease, who has intriguing endoscopic findings. Upon initial diagnosis at age 20, he received steroid therapy, but has not required any further medical intervention. He has remained relatively asymptomatic and keeps a healthy lifestyle. At routine colonoscopy, we identified pseudopolyps as well as tissue bridges within the colon, giving an unusual “swiss cheese” appearance. This case exemplifies the heterogeneity of Crohn’s disease, emphasizing the possibility of finding evidence of ongoing disease despite lack of symptoms.

A 64-year-old man with a 44-year history of Crohn’s disease presented for a routine colonoscopy. The patient’s past medical treatment was a short course of steroid therapy at the initial time of diagnosis but he has not been on maintenance medication since then. The patient has a normal appetite but avoids red meat and alcohol, which can cause bloating. Currently, the patient denies other common symptoms, such as abdominal pain, diarrhea, or weight loss. Routine colonoscopy demonstrated marked mucosal abnormality within the descending, sigmoid, and ascending colon, characterized by tissue bridges, giving a “Swiss cheese” appearance (Figure 1). At one point during the procedure, visualization of the proximal aspect of the colonoscope was possible through the adjacent communicating colonic walls. Pseudopolyps were found within the sigmoid and ascending colon/splenic flexure and these were biopsied (Figure 2). Microscopic examination of the pseudopolyps revealed features of inflammatory polyps (Figure 3). The patient continues to lead a healthy lifestyle and requires no further medical management.

**Figure 1.**
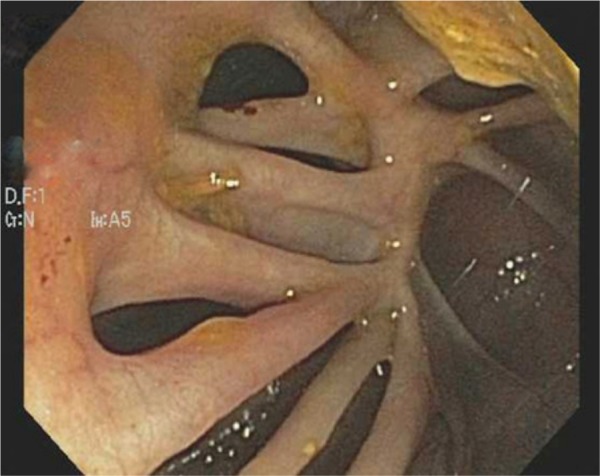
“Swiss cheese” appearance of the sigmoid colon is seen here. During the procedure there were similar appearances in the descending and ascending colon (colonoscopic image).

**Figure 2.**
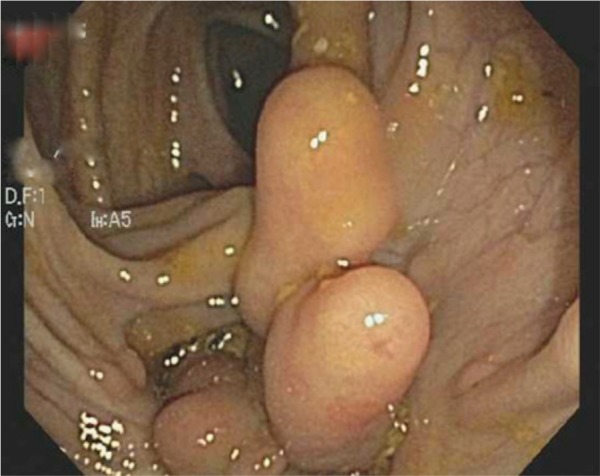
Localized pseudopolyps were found and biopsied in the sigmoid and ascending colon/splenic flexure (colonoscopic image).

**Figure 3.**
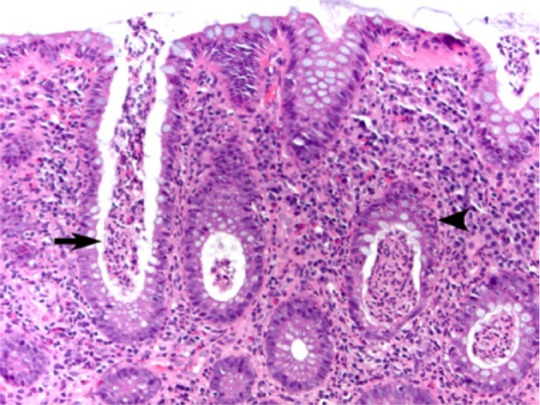
Histologic section of biopsy tissue obtained from a pseudopolyp shows cryptitis (arrowhead) and crypt abscesses (arrow), consistent with inflammatory polyp (hematoxylin and eosin stain, original magnification 20×).

Crohn’s disease is extremely heterogeneous, and patients may present with endoscopic and histologic findings consistent with persistent disease, despite being asymptomatic for decades. A recent study reported evidence of inflammation either by endoscopy or histology in 31% of patients with asymptomatic Crohn’s disease.^1^ Although attempts have been made to predict severity of the disease, including a number of severity indices, variations persist, and optimal disease monitoring strategies incorporating several disease scoring systems have only recently been determined in a consensus setting.^2^ Transmural inflammation can manifest as strictures, fistulas, and ulceration and can lead to abscess formation. The diffuse “Swiss cheese” appearance identified here is consistent with chronic mucosal injury, and the finding of inflammatory polyps on histologic evaluation is indicative of an ongoing active disease process. In this case, however, the lack of symptoms despite the intriguing endoscopic findings has led to a relatively benign disease course, and the patient will continue to be followed without additional medical therapy.
